# Infoxicación en salud. La sobrecarga de información sobre salud en la web y el riesgo de que lo importante se haga invisible

**DOI:** 10.26633/RPSP.2017.115

**Published:** 2017-09-29

**Authors:** Marcelo D´Agostino, Felipe Medina Mejía, Myrna Martí, David Novillo-Ortiz, Flavio Hazrum, Federico G. de Cosío

**Affiliations:** 1 Organización Panamericana de la Salud, Unidad de Información y Análisis de Salud Departamento de Enfermedades Transmisibles Washington, DC. Estados Unidos de América Organización Panamericana de la Salud, Unidad de Información y Análisis de Salud, Departamento de Enfermedades Transmisibles, Washington, DC, Estados Unidos de América.; 2 Salud Pública y Redes Sociales Salud Pública y Redes Sociales Bogotá Colombia Salud Pública y Redes Sociales, Bogotá, Colombia.; 3 Organización Panamericana de la Salud Oficina de Gestión del Conocimiento, Bioética e Investigación Buenos Aires Argentina Organización Panamericana de la Salud, Oficina de Gestión del Conocimiento, Bioética e Investigación, Buenos Aires, Argentina.

**Keywords:** Centros de información, confidencialidad, servicios de información, teoría de la información, informática médica, presentación de datos, almacenamiento y recuperación de la información, y ciencia de la información, Information centers, confidentiality, information services, information theory, medical informatics, data display, information storage and retrieval, information science, Centros de informação, confidencialidade, serviços de informação, teoria da informação, informática médica, apresentação de dados, armazenamento e recuperação da informação, ciência da informação

## Abstract

**Objetivo.:**

*Este estudio tiene como objetivos 1) crear conciencia del volumen de información en salud existente en la web de calidad, 2) explorar la percepción de profesionales de la información con relación al uso de fuentes cualificadas en la toma de decisiones en salud, y 3) presentar recomendaciones que permitan fortalecer las capacidades de los trabajadores de la salud y las competencias institucionales relacionadas con la alfabetización digital*.

**Métodos.:**

Se realizó un estudio no experimental descriptivo transversal con una muestra no probabilística de 32 profesionales de la información de nueve países. Se recopiló información de internet sobre el volumen de contenidos existentes en herramientas web, redes sociales y fuentes de información en salud. Se realizaron búsquedas en inglés y en español utilizando las palabras clave Ebola, Zika, Dengue, Chikungunya, Safe food, Health equity, Safe sex, y Obesit. Por último, se obtuvo información sobre la oferta de formación formal en temas de alfabetización digital, gestión de información y otros relacionados.

**Resultados.:**

Seleccionando sólo cuatro enfermedades de alto impacto en salud pública en mayo de 2016 y promediando un tiempo de revisión mínimo de cada producto de información, se tardaría más de 50 años seguidos sin dormir para consultar todo lo publicado en línea sobre Dengue, Zika, Ebola y Chikungunja.

**Conclusión.:**

*Se concluye que la salud pública se beneficiaría con instituciones de salud que implementaran estrategias formales de gestión del conocimiento, con instituciones académicas de ciencias de la salud que incorporaran programas formales de alfabetización digital y con trabajadores de la salud cuyo desarrollo profesional sea responsable y funcional en la sociedad de la información*.

## Abstract (en)

**Objective.:**

The objectives of this study were to: 1) raise awareness of the volume of quality health information on the Internet; 2) explore perceptions of information professionals with regard to the use of qualified sources for health decision-making; and 3) make recommendations that facilitate strengthening health worker capacities and institutional competencies related to digital literacy.

**Methods.:**

A non-experimental, descriptive cross-sectional study was conducted with a non-probability sample of 32 information professionals from nine countries. Internet information was compiled on the volume of content in Internet tools, social networks, and health information sources. Searches in English and Spanish were carried out using the keywords Ebola, Zika, dengue, chikungunya, safe food, health equity, safe sex, and obesity. Finally, information was obtained on opportunities for formal education on the subjects of digital literacy, information management, and other related topics.

**Results.:**

Selecting only four diseases with a high impact on public health in May 2016 and averaging minimum review time for each information product, it would take more than 50 years without sleeping to consult everything that is published online about dengue, Zika, Ebola, and chikungunya.

**Conclusion.:**

We conclude that public health would benefit from: health institutions implementing formal knowledge management strategies; academic health sciences institutions incorporating formal digital literacy programs; and having health workers who are professionally responsible and functional in the information society.

## RESUMO

**Objetivos.:**

1) Conscientizar sobre o volume de informação em saúde de qualidade existente na internet, 2) explorar a percepção dos profissionais da informação sobre o uso de fontes qualificadas na tomada de decisão em saúde e 3) fazer recomendações para reforçar o preparo dos profissionais de saúde e as competências institucionais quanto à alfabetização digital.

**Métodos.:**

Foi realizado estudo transversal descritivo não experimental em uma amostra não probabilística com 32 profissionais da informação de nove países. Foi coletada informação da internet sobre o volume de conteúdos existentes sobre ferramentas de internet, redes sociais e fontes de informação em saúde. Foram realizadas buscas em inglês e espanhol utilizando as palavras-chave *Ebola, Zika, Dengue, Chikungunya, Safe food, Health equity, Safe sex, e Obesity*. E foi obtida informação sobre a oferta de cursos de formação em alfabetização digital, gestão da informação e outros tópicos relacionados.

**Resultados.:**

Com a seleção de apenas quatro doenças de alto impacto em saúde pública em maio de 2016, com tempo de revisão médio mínimo para cada produto de informação, seriam necessários mais de 50 anos contínuos, sem intervalo para dormir, para consultar todo o conteúdo publicado online sobre dengue, zika, Ebola e chikungunya.

**Conclusão.:**

Concluise que existiria benefício para a saúde pública se as instituições de saúde implementassem estratégias formais de gestão do conhecimento, as instituições acadêmicas de ciências da saúde incorporassem programas formais de alfabetização digital e os profissionais de saúde investissem em desenvolvimento profissional responsável e prático na sociedade da informação.

La preocupación por la cuantificación del volumen de información existente comenzó en el siglo XIX cuando se incrementaron el uso y la recopilación de datos estadísticos como base para los debates públicos y la formulación de políticas. Las comparaciones internacionales proliferaron a juzgar por estadísticas como la circulación de diarios, la venta de libros y el tamaño de las bibliotecas (1). En la actualidad, la situación se torna más compleja con el auge de los contenidos sociales que circulan en internet y que no están clasificados ni estructurados. Se barajan algunas hipótesis sobre el valor del contenido no estructurado diseminado en las redes sociales y su potencial beneficio en salud pública (2), sustentadas por alguna evidencia de que estas redes, que se presentan como fuentes masivas de contenidos, podrían brindar oportunidades para apoyar diversas intervenciones en salud pública (3-6). Sin embargo, para llegar a un mejor entendimiento del valor de la información que circula por las redes sociales, se requiere una comprensión cabal sobre el comportamiento en línea de los individuos y sobre el uso de las tecnologías y la información (7).

Los movimientos relacionados con el concepto “apertura de contenidos” ganaron fuerza con internet y el uso masivo de las tecnologías de la información y la comunicación (TIC). La Relatoría Especial para la Libertad de Expresión de la Comisión Interamericana de Derechos Humanos afirma que el acceso a la información constituye una herramienta esencial para combatir la corrupción, hacer realidad el principio de transparencia en la gestión pública y mejorar la calidad de las democracias (http://www.oas.org/es/cidh/expresion/index.asp). Por otro lado, la Organización Mundial de la Salud (OMS) hace referencia a la importancia de promover y proteger el derecho a la educación y el derecho a buscar, recibir y difundir informaciones e ideas relativas a las cuestiones de salud y deja claro que el derecho a la información no debe menoscabar el derecho a la intimidad, lo que significa que debe darse un trato confidencial a los datos personales relativos a la salud (http://www.who.int/hhr/activities/Q%26AfinalversionSpanish.pdf). El 27 de mayo de 2016, la Unión Europea acordó trabajar con el ambicioso objetivo de dar libre acceso a todos los trabajos científicos para 2020 (https://ec.europa.eu/programmes/horizon2020/sites/horizon2020/files/H2020_ES_ KI0213413ESN.pdf). A pesar de ser una buena noticia, es importante resaltar que aún quedan por resolverse los temas de financiamiento de esta propuesta. Muchas de las revistas científicas en la actualidad cobran para que un artículo sea de libre acceso.

En el presente artículo se plantean los siguientes objetivos: 1) crear conciencia del volumen de información en salud existente en la web de calidad, 2) explorar la percepción de profesionales de la información con relación al uso de fuentes calificadas en la toma de decisiones en salud, y 3) presentar recomendaciones que permitan fortalecer las capacidades de cualquier trabajador de la salud y las competencias institucionales relacionadas con la alfabetización digital. Considerando que los trabajadores y las instituciones de salud, así como la población general, son muy activos en la creación e interacción de los contenidos en línea, y que existe un crecimiento exponencial de información estructurada y no estructurada, también se analiza el qué, quién, para quién y cómo crear nuevos contenidos sólo cuando sea necesario.

## MATERIALES Y MÉTODOS

Se realizó un estudio no experimental, descriptivo y transversal con una muestra no probabilística de 32 profesionales de la información de Argentina, Bolivia, Brasil, Chile, Colombia, Estados Unidos de América, México, Panamá y Venezuela y se analizaron datos extraídos de internet sobre el volumen de contenidos existentes en herramientas web, redes sociales, fuentes de información en salud y la oferta de formación académica y profesional por parte de instituciones. El criterio de inclusión de los integrantes de la muestra fue que fueran profesionales de la información, bibliotecarios y especialistas en salud pública.

Con el objetivo de conocer la percepción de quienes trabajan en centros de información de instituciones de salud pública acerca del uso directo o indirecto de fuentes de información de calidad por parte de los que toman decisiones o trabajadores de salud pública, se diseñó un cuestionario en línea de 10 preguntas cerradas con posibilidad de incluir comentarios abiertos. El cuestionario se distribuyó a distintas redes de información en salud de bibliotecas de la Región de las Américas y las respuestas fueron de carácter anónimo.

Para recopilar la información que hay en internet sobre el volumen de contenidos existentes en herramientas web, redes sociales y fuentes de información en salud, se realizaron búsquedas tanto en inglés como en español utilizando las siguientes palabras clave: Ebola, Zika, Dengue, Chikungunya, Safe food, Health equity, Safe sex, y Obesit. Como herramientas web y redes sociales se seleccionaron las siguientes: el motor de búsqueda de Google, noticias de búsqueda de Google, Google Videos, Google Books, Youtube, Twitter, Slideshare, y LinkedIn. Las fuentes de información científico-técnica seleccionadas para el estudio fueron PubMed, PubMed Central, Medline Plus, Cochrane Library, Google Scholar, Healthfinder, Redalyc, y la Biblioteca Virtual en Salud de BIREME/OPS/OMS. BIREME es el Centro Latinoamericano y del Caribe de Información en Ciencias de la Salud adscrito a la Oficina de Gestión del Conocimiento, Bioética e Investigación de la OPS/OMS. Para recabar la información sobre la oferta de formación formal en temas de alfabetización digital, gestión de información o manejo de búsquedas avanzadas de información en salud y carreras relacionadas con las ciencias de la salud, se realizó una búsqueda de cursos formales o materias específicas relacionadas con el concepto de alfabetización digital, gestión de información o manejo de búsquedas avanzadas de información en salud. Con esta finalidad, se utilizaron las siguientes estrategias de búsquedas en dos idiomas: [“Ciencias de la Información” AND “Salud pública” AND “Facultad”], [“Alfabetización digital” AND “Salud Pública” AND “Facultad”], [“Búsquedas avanzadas” AND “Salud Pública” AND “Facultad”], [“Alfabetización informacional” AND “Salud Pública” AND “Facultad”], [“Health Science Information” AND “Public Health” AND “Faculty”], [“Digital literacy” AND “Public Health” AND “Faculty”], [“Advances searches” AND “Public Health” AND “Faculty”], y [“Information literacy” AND “Public Health” AND “Faculty”].

## RESULTADOS

**Herramientas web y redes sociales.** El análisis de la información en internet sobre el volumen de contenidos existentes en herramientas web y redes sociales (cuadro 1) revela que a mayo de 2016 YouTube notificó que tenía más de mil millones de usuarios (lo que equivaldría a un tercio de todos los usuarios de internet) y destaca que cada día se ven cientos de millones de horas de vídeos y se generan miles de millones de reproducciones. El tiempo de visualización en YouTube ha aumentado al menos 50% entre años durante tres años consecutivos. Por otro lado, Google procesa más de 50 000 búsquedas por segundo en promedio, lo que se traduce en más de 35 millones de búsquedas al día en todo el mundo. En Twitter, cada segundo en promedio se producen alrededor de 6 000 tweets, lo que corresponde a más de 350 000 contenidos compartidos por minuto, 500 millones de tweets diarios y alrededor de 200 mil millones de tweets por año (http://www.internetlivestats.com/).

**CUADRO 1.** Contenido existente en las herramientas sociales a 20 de marzo de 2016

Ebola	Zika	Dengue	Chikungunya	Safe food	Health equity	Safe sex	Obesity
Youtube	1 680 000	1 040 000	1 060 000	200 000	16 800	13 700	93 700	679 000
Slideshare	4 542	526	7 477	1 514	2 149	1 414	1 516	50 034
LinkedIn	19 448	1 591	14 745	1 739	15 168	15 078	5 121	95 645
Google web search	55 800 000	108 000 000	56 100 000	10 300 000	731 000	776 000	8 630 000	77 000 000
Google news	11 300 000	41 400 000	3 600 000	487 000	27 100	18 700	61 400	1 690 000
Google videos	8 720 000	1 350 00	685 000	284 000	29 900	26 900	396 000	1 710 000
Google books	158 000	91 600	464 000	37 900	82 000	36 600	168 000	687 000
Total	77 681 990	150 533 717	61 931 222	11 312 153	904 117	888 392	9 355 737	81 911 679

***Fuente:*** Internet.

### Conocimiento de conceptos relacionados con las ciencias de la información.

Cuando los encuestados fueron consultados sobre qué conceptos creían que eran conocidos por los profesionales de la salud, apenas 3% consideró que el de “gestión de contenidos” es entendido por estos profesionales. Sin embargo, hay una tendencia a pensar que se trata de un concepto demasiado amplio y, por tanto, que no es relevante su total comprensión. Existe la impresión de que la gestión de contenidos se sigue viendo como algo que otros hacen cuando suben información a la página web de una institución.

**FIGURA 1.**
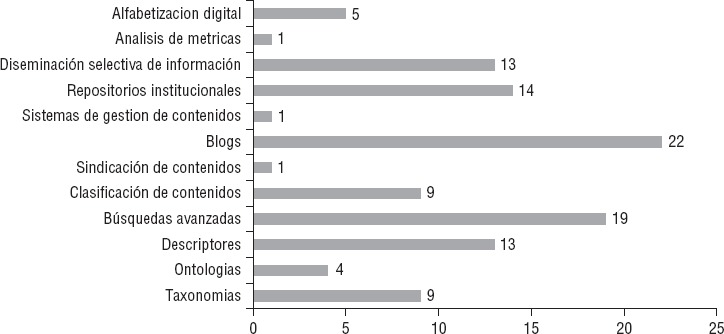
Respuesta a la pregunta: Marque los conceptos que usted considera que son conocidos por los trabajadores de la salud

Con relación a otros conceptos más específicos (figura 1), se pone de manifiesto la irrelevancia de que los que toman decisiones sepan qué es una taxonomía, una ontología o incluso un tesauro o un término controlado. Sin embargo, se destaca la importancia de saber que existen herramientas para la normalización e indexación de contenidos que actúan como vías resolutivas para la confección de búsquedas específicas.

### Conocimiento de la existencia y forma de uso de las fuentes de información científica y técnica en salud.

Sobre el conocimiento y el uso regular o irregular de información científica y técnica para la toma de decisiones existe una percepción positiva, aunque se subraya la importancia de entender los diferentes tipos de toma de decisión que se realizan en una institución de salud. Quienes están en la dirección y en puestos de alta gerencia tiene perfiles de gestión, no disponen de todo el tiempo necesario y la consulta de la bibliografía científica no es requerimiento del perfil que ocupan.No obstante, en general cuentan con equipos que preparan resúmenes para ellos. Es importante observar que casi 90% de ellos reconoce que los que toman decisiones están familiarizados con las fuentes de información de mayor cali-dad del mundo en ciencias de la salud,así como con la mayoría de las fuentes especializadas. En respuesta a la pregunta sobre si quienes toman decisiones consultan o no la bibliografía científica o técnica regularmente, los profesionales de la información encuestados piensan que más de 50% no consultan directamente dicha bibliografía de estas fuentes, lo cual puede deberse a que las decisiones están fundamentadas en información analizada por equipos técnicos que tienen un perfil eminentemente de investigación o técnico o de generación de síntesis de información sobre temas específicos.

***Fuente:*** cuestionario desarrollado para el estudio.

**FIGURA 2.**
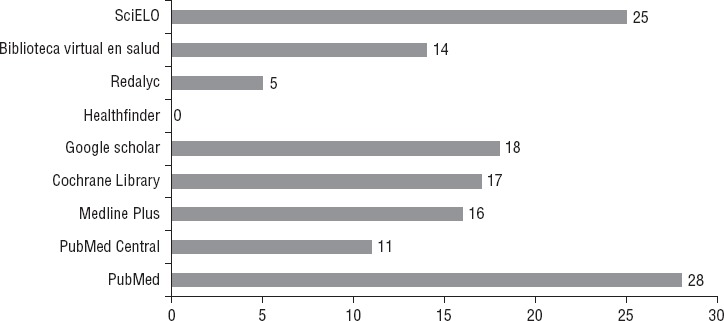
Respuesta a la pregunta: Considera usted que los trabajadores de la salud conocen la existencia de estos recursos de la información

***Fuente:*** cuestionario desarrollado para el estudio.

Respecto a los motores de búsqueda de las publicaciones periódicas y fuentes de información especializadas se destaca que, en general, son del dominio profesional de los bibliotecnólogos y no de uso masivo. Por ello es importante reforzar y formalizar los vínculos que existen entre los que toman decisiones y los profesionales de la información de las instituciones. Se destaca, además, la importancia de capacitar a los decisores y a los trabajadores de la salud en la definición del alcance de las búsquedas más que en las búsquedas en sí mismas. Al usar la herramienta Pubmed, se observa que los usuarios tienen algunas dificultades con la implementación de filtros de búsquedas y con el reconocimiento de resultados específicos. Asimismo, y a partir del análisis realizado por uno de los profesionales encuestados, evaluando los 5 000 usuarios de su red a los cuales se les ofrecen tutoriales para el desarrollo de búsquedas de información, en la mayoría de los casos terminan solicitando el material a la biblioteca. Por otro lado, se considera que los que realizan actividades de investigación cuentan con un desarrollo de excelencia de estas habilidades de búsqueda, lo que les ayuda a encontrar la evidencia científica necesaria para la toma de decisiones.

### Contenidos en herramientas sociales.

Según el cuadro 1, si se considera que el tiempo promedio de duración de un video de Youtube es de 5 minutos, para que una persona pueda ver sólo aquellos videos sobre ébola que existen en esta red social, se necesitarían 140 000 horas, lo que equivale a 5 800 días o 16 años seguidos sin dormir. Si, además de esto, esa misma persona es responsable de las enfermedades transmisibles, también sería responsable de dar respuesta sobre el Dengue, el Zika y la Chikungunya. Esto significa que pasaría 331 667 horas revisando contenidos ya existentes, es decir, 13 819 días o 38 años seguidos sin dormir. Y si, además, se le añaden los textos, audios, documentos y otros materiales que existen en todas las herramientas sociales y las científico-técnicas superaría los 50 años seguidos sin dormir.

### Contenidos en fuentes de información en salud.

A diferencia de los contenidos sociales, los que contienen las bases de datos científicas y técnicas de calidad son el resultado de una revisión por pares. Este proceso es un método que se utiliza para validar trabajos científicos. Su propósito principal es medir la calidad de las investigaciones que se intentan publicar. Si bien es un proceso respetado y considerado valioso, uno de los aspectos más criticados es su lentitud (8, 9). En el cuadro 2 puede apreciarse el volumen de contenidos existente por cada tema en cada una de las fuentes de información cualificadas que fueron exploradas para el presente estudio.

### Formación académica profesional.

Los resultados de la búsqueda de oferta académica en internet sobre formación académica profesional en alfabetización digital indican que, si bien existen algunos cursos, materias o elementos de aprendizaje sobre temas de gestión de información, alfabetización digital y otros conceptos relacionados, la gran mayoría de las facultades de medicina o escuelas de postgrado de salud pública de la Región no disponen de programas formales de educación relacionados con estos temas. Una excepción y buen ejemplo de ello, que no es lo habitual en el mundo académico de las ciencias de la salud, puede verse en la Universidad de Harvard, que mantiene un sitio web llamado Health Literacy Website (http://www.hsph.harvard.edu/healthliteracy/) diseñado por profesionales de la salud y de la educación interesados en estos temas. También es importante reconocer en esta línea la existencia de iniciativas de organismos internacionales, como los cursos desarrollados por la Organización Panamericana de la Salud (OPS) de “Acceso a fuentes de información y uso de redes sociales” disponible en el Campus Virtual de Salud Pública (https://www.campusvirtualsp.org/es/curso-accesofuentes-de-informacion-y-manejo-de-redes-sociales), así como una serie de iniciativas presenciales y virtuales gestionadas por el Centro Latinoamericano y del Caribe de Información en Ciencias de la Salud (BIREME) sobre acceso a información en salud. Con ambas iniciativas ya se han formado miles de trabajadores de la salud de toda la Región.

## DISCUSIÓN

Es importante hacer una reflexión a partir de dos ejes conceptuales. El primero es el relacionado con el volumen de contenidos existentes, un tema que preocupa a la humanidad desde, al menos, 300 años antes de Cristo cuando se creó la Biblioteca de Alejandría (1). En relación con el tema que corresponde a este análisis, y respecto al contenido que se encuentra disponible en formato digital, los resultados concuerdan con lo expresado por Andrew Odylzco cuando, preocupado por el volumen y el valor de la información y respecto a la capacidad y el espacio de almacenamiento, advirtió del gran volumen de información que se produce a diario (1).

**CUADRO 2.** Contenido de las fuentes de información cualificadas a 20 de marzo de 2016

Ébola	Zika	Dengue	Chikungunya	Safe food	Health equity	Safe sex	Obesity
PubMed	5 072	463	15 128	2698	452	2 710	3 264	236 097
PubMed Central	7 407	374	19 980	3 681	882	4 652	3 348	190 290
Medline Plus	185	1 101	90	34	83	966	76	2 156
Cochrane Library	25	0	279	15	6	94	417	18 680
Google scholar	195 000	26 500	387 000	27 300	33 300	41 600	47 800	1 980 000
Healthfinder	19 448	1 591	14 745	1 739	15 168	15 078	5 121	473
Redalyc	259	144	4 389	89	103	356	250	7 816
Virtual Health	4 956	245	19 041	2 621	404	1 094	2 987	221 756
Library	
Total	232 352	30 418	460 652	38 177	50 398	66 550	63 263	2 657 268

***Fuente:*** internet.

En este sentido, el presente estudio añade un foco hacia el entendimiento y el análisis de los contenidos, que, según los resultados, es imposible analizar en su totalidad, principalmente por los que toman decisiones y los trabajadores de la salud, quienes, además de no tener el tiempo necesario, carecen de la formación adecuada para dominar los métodos y las herramientas requeridos para acceder a información útil en el momento y con el formato oportunos. Además, es importante entender la frecuencia de actualización de los contenidos, estructurados y no estructurados, y cómo esto está afectando a la capacidad de encon-trar lo que se busca cuando se necesita y en el formato más apropiado. De esta manera, la gran cantidad de información disponible, combinada con la falta de capacidades de búsqueda, puede producir decisiones incorrectas tanto a la hora de buscar como al elaborar nuevos contenidos, lo que resulta en una innecesaria inversión de recursos financieros y humanos y en la sobrecarga añadida de la web con contenidos que no necesariamente deberían desarrollarse.

En la *sociedad de la información* (un concepto acuñado y divulgado por Yoneji Masuda, que comenzó a utilizarse en Japón durante los años sesenta), los trabajadores de la salud, las instituciones relacionadas con la salud y la población general son muy activos en la creación e interacción con los contenidos en línea, tanto estructurados como no estructurados.

**FIGURA 3.**
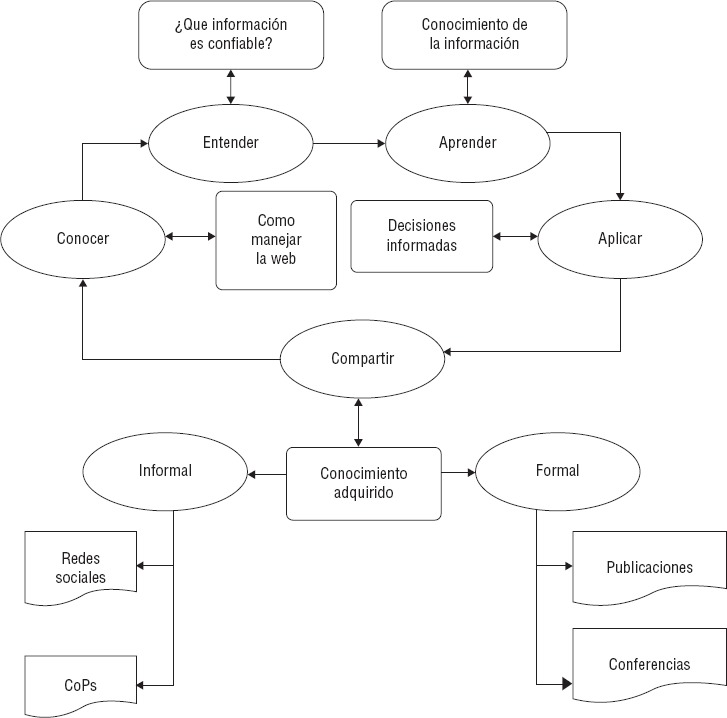
El ciclo de la responsabilidad de las personas en la Sociedad de la*información*: Conocer, Entender, Aprender, Aplicar y Compartir el conocimiento

En concordancia con el Informe sobre Big Data y Salud del Instituto Tecnológico de Massachusetts (3), el análisis realizado permite apreciar que la calidad de los contenidos de las bases de datos científicas y técnicas, a pesar de ser revisados por pares, se ve afectada por su gran volumen, lo que plantea el mismo desafío que los contenidos sociales, ya que, como muestran los resultados, se considera imposible poder analizar todo la informa-ción disponible y discernir lo que podría ser útil y necesario en el momento y formato adecuado con calidad, confianza y veracidad. Por otro lado, no existe plena conciencia sobre la responsabilidad que hoy tiene un trabajador de la salud con acceso a información de entender los flujos de información, diseminación y comunicación que se plantean en la sociedad de la información y requieren su participación, como muestra la figura 3.

El acceso a información cualificada debe pasar de ser un beneficio individual a una responsabilidad social. Es importante entender que, para que exista información de calidad publicada y disponible de manera libre y abierta, todos los trabajadores de la salud, en mayor o menor medida, deben compartir lo que saben y lo que aprenden diariamente. Esto es un paso necesario y un compromiso social para que aquellos que tienen menos acceso al conocimiento y a las tecnologías de información se beneficien de los que sí lo tienen (10).

***Fuente:***http://marcelodagostino.wordpress.com

Siguiendo el debate iniciado por Odylzco (1), los resultados de este estudio indican que no sólo es el espacio de almacenamiento el recurso limitado que puede agotarse con la producción constante, sino que el problema planteado se relaciona con la falta de estrategias formales y de una cultura organizativa que promuevan este tipo de análisis.

Esta última observación introduce el segundo eje conceptual: la importancia de implantar mecanismos institucionales para definir qué, quién, para quién, cómo y cuándo crear nuevos contenidos, sólo cuando sea necesario, y optimizando tiempo y recursos humanos y financieros. Si se necesitan 38 años para ver los videos existentes en Youtube sobre Dengue, Ebola, Zyka y Chicungunha, se plantea la necesidad de que las instituciones revisen y creen sus estrategias, cultura o costumbre de eliminar contenidos publicados en línea utilizando parámetros como su relevancia con procesos formales que permitan a los trabajadores de la salud revisar y analizar contenidos antes de tomar la decisión de producir nuevos.

Conocer las limitaciones de la calidad del contenido de las redes sociales, como afirman Paul y Dredze (6), es una habilidad necesaria para los profesionales que intentan acceder al contenido disponible. Asimismo, si a los contenidos no estructurados de las redes sociales se suman la cantidad de contenidos técnicos y científicos existentes y accesibles en bases de datos calificadas, nos encontramos ante una encrucijada difícil de resolver que debe analizarse: existencia de contenidos disponibles que no logramos conocer por la falta de conocimiento para buscarlos o, en otras palabras, contenidos que sencillamente no existen para quienes no saben encontrarlos. Y, por consiguiente, nace una importante y peligrosa contradicción: a mayor cantidad de contenidos existentes y accesibles, menor es la probabilidad de encontrar lo que se necesita cuando se necesita y en el formato que se necesita para tomar una decisión adecuada y oportuna. Dicha encrucijada y contradicción en el ámbito institucional lleva a tomar decisiones incorrectas de producir nuevos contenidos, tal vez innecesarios, generalmente haciendo una inversión inadecuada de recursos humanos y financieros en lugar de usar, comentar, criticar o mejorar los contenidos ya existentes. Por otro lado, comprender el valor del contenido no estructurado en línea implica necesariamente implantar mecanismos para entender el comportamiento social de los que navegan en la web.

La mayor limitación de este estudio es que la forma en que las instituciones académicas presentan los cursos de formación citados no está estandarizada y que los denominan e interpretan de diversas formas. Como el número de encuestas respondidas puede no resultar muy significativo ni representativo para países de América Central y el Caribe, se recomienda aplicar la misma herramienta en estos países para aumentar la representatividad del estudio.

En la sociedad de la información es fundamental tener habilidades profesionales para identificar los contenidos necesarios, para las personas adecuadas y en el momento y el formato oportunos. Para ello es necesario promover y fortalecer estrategias de gestión del conocimiento y proyectos de fortalecimiento de la cultura organizativa que subrayen la importancia de estos temas y a un tiempo implementar normas y procedimientos para que la toma de decisiones y la formulación de políticas a todos los niveles se realice sobre la base de evidencias científicas y técnicas. El desarrollo de una estrategia de gestión del conocimiento, que incluya un programa de alfabetización digital, permitirá comprender mejor los contenidos que hoy están disponibles y son accesibles e implantar mecanismos institucionales para tomar decisiones costo-efectivas acerca de qué, quién, para quién, cómo y cuándo crear nuevos contenidos, sólo cuando sea necesario.

Un buen ejemplo de ello es la estrategia de gestión del conocimiento que el Ministerio de Salud de Dominica (11) está desplegando partir de la estrategia de gestión del conocimiento de la OPS(12) con la finalidad de apoyar y fortalecer la agenda nacional de salud del país. Acciones formales en los sistemas de salud que mejoren las capacidades individuales y colectivas sobre búsquedas, gestión y diseminación de información traerán como resultado mejores políticas y acciones más efectivas que beneficien a la población, así como pacientes más y mejor informados. Un estudio de 2015 del Pew Research Center ((http://www.pewinternet.org/2015/09/15/libraries-at-the-crossroads/) reveló que “el 73% de personas mayores a 16 años dicen que las bibliotecas contribuyen con la población para encontrar la información de salud que necesitan. El 42% de las personas que respondieron mencionan que lo han hecho para hacer búsquedas relacionadas con temas salud”. En 2013, el Proyecto Pew Internet Research informó de que “el 59% de los adultos estadounidenses dicen que han buscado en línea información sobre una variedad de temas de salud y 35% de ellos dicen que han buscado específicamente para tratar de averiguar sobre algún tipo de problema médico propio o de alguna otra persona” (13).

Por otro lado, la falta de estrategias de gestión del conocimiento puede desembocar en la provisión o en el consumo de información inadecuada y en un uso ineficaz de los recursos financieros y humanos asignados a la salud pública.

Es crítico que las instituciones académicas relacionadas con las ciencias de la salud incorporen formalmente programas académicos de alfabetización digital y algunas normas para la alfabetización informacional en la educación superior, como las desarrolladas por la Association of College and Research Libraries o las de la Society of College National and University Libraries, que describen un modelo de habilidades en información en la educación superior que estimula el pensamiento crítico, la información como insumo para la adopción de decisiones y la generación de conocimientos (14-16).

Desde el punto de vista del uso de tecnologías de la información, es crítico que se avance en desarrollos de software y aplicaciones para procesar y analizar conjuntamente datos estructurados y no estructurados, compuestos por bases de datos, textos, audios, videos, bibliografía científica y técnica, y compararlos con el comportamiento social de las personas en línea.

Es crucial también reforzar y formalizar los vínculos que existen entre los que toman decisiones y los profesionales de la información de las instituciones, así como capacitarlos, junto con los trabajadores de salud, en la definición del alcance de las búsquedas más que en las búsquedas en sí mismas.

Por último, la salud pública se beneficiaría con instituciones de salud que implanten estrategias formales de gestión del conocimiento, con instituciones académicas de ciencias de la salud que incorporen programas formales de alfabetización digital, y con trabajadores de la salud cuyo desarrollo profesional sea responsable y funcional en la sociedad de la información.

### Agradecimiento.

Este trabajo fue realizado con el apoyo de bibliotecarias y bibliotecarios responsables de bibliotecas especializadas en ciencias de la salud de Argentina, Bolivia, Brasil, Chile, Colombia, Estados Unidos de América, México, Panamá y Venezuela.

### Financiación.

Este estudio no recibió financiación.

### Declaración.

Las opiniones expresadas por los autores son de su exclusiva responsabilidad y no reflejan necesariamente los criterios ni la política de la Organización Panamericana de la Salud o de la RPSP/PAJPH.
